# The cholesteryl-ester transfer protein isoform (CETPI) and derived peptides: new targets in the study of Gram-negative sepsis

**DOI:** 10.1186/s10020-022-00585-3

**Published:** 2022-12-19

**Authors:** Eréndira G. Pérez-Hernández, Víctor De la Puente-Díaz de León, Ismael Luna-Reyes, Blanca Delgado-Coello, José Sifuentes-Osornio, Jaime Mas-Oliva

**Affiliations:** 1 Instituto de Fisiología Celular, Universidad Nacional Autónoma de México, 04510 Ciudad de Mexico, Mexico; 2 Departamento de Medicina Interna, Instituto Nacional de Ciencias Médicas y Nutrición “Salvador Zubirán”, 14080 Ciudad de Mexico, Mexico; 3 Dirección de Medicina, Instituto Nacional de Ciencias Médicas y Nutrición “Salvador Zubirán”, 14080 Ciudad de Mexico, Mexico

**Keywords:** CETPI, LPS, Gram-negative bacteria, Sepsis, Septic shock, CETP

## Abstract

**Background:**

Sepsis is a syndrome where the dysregulated host response to infection threatens the life of the patient. The isoform of the cholesteryl-ester transfer protein (CETPI) is synthesized in the small intestine, and it is present in human plasma. CETPI and peptides derived from its C-terminal sequence present the ability to bind and deactivate bacterial lipopolysaccharides (LPS). The present study establishes the relationship between the plasma levels of CETPI and disease severity of sepsis due to Gram-negative bacteria.

**Methods:**

Plasma samples from healthy subjects and patients with positive blood culture for Gram-negative bacteria were collected at the Intensive Care Unit (ICU) of INCMNSZ (Mexico City). 47 healthy subjects, 50 patients with infection, and 55 patients with sepsis and septic shock, were enrolled in this study. CETPI plasma levels were measured by an enzyme-linked immunosorbent assay and its expression confirmed by Western Blot analysis. Plasma cytokines (IL-1β, TNFα, IL-6, IL-8, IL-12p70, IFNγ, and IL-10) were measured in both, healthy subjects, and patients, and directly correlated with their CETPI plasma levels and severity of clinical parameters. Sequential Organ Failure Assessment (SOFA) scores were evaluated at ICU admission and within 24 h of admission. Plasma LPS and CETPI levels were also measured and studied in patients  with liver dysfunction.

**Results:**

The level of CETPI in plasma was found to be higher in patients with positive blood culture for Gram-negative bacteria that in control subjects, showing a direct correlation with their SOFA values. Accordingly, septic shock patients showing a high CETPI plasma concentration, presented a negative correlation with cytokines IL-8, IL-1β, and IL-10. Also, in patients  with liver dysfunction, since higher CETPI levels correlated with a high plasma LPS concentration, LPS neutralization carried out by CETPI might be considered a physiological response that will have to be studied in detail.

**Conclusions:**

Elevated levels of plasma CETPI were associated with disease severity and organ failure in patients  with Gram-negative bacteraemia, defining CETPI as a protein implicated in the systemic response to LPS.

**Supplementary Information:**

The online version contains supplementary material available at 10.1186/s10020-022-00585-3.

## Background

Each year an elevated number of people worldwide experience an episode of sepsis, where millions die as a result of its associated complications (Singer et al. 2016; Reinhart et al. 2017). In low- and middle-income countries, the burden of sepsis is even higher and represents one of the leading causes of death in the general population, where a complex interaction of factors such as the causative pathogen, timely diagnosis, host immunity, and access to quality care, determine lethality in sepsis (Cecconi et al. 2018; Rudd et al. 2020). Sepsis occurs when the host produces an unbalanced response to an infection, that if not diagnosed and treated early, can lead to profound circulatory and cellular dysfunction, progressing to septic shock (Cecconi et al. 2018). Gram-negative bacteria are the most frequent etiological agents of sepsis worldwide (Font et al. 2020), presenting in their outer membrane LPS. The physiological response to LPS is mediated by stimulation of the Toll-like receptor 4, that includes the release of pro-inflammatory cytokines and reactive oxygen species, causing endothelial damage and vasodilation, leading to hypo-perfusion and capillary fluid leakage. Additionally, cytokines activate the coagulation cascade, resulting in capillary microthrombi, and ultimately organ ischemia (Van Der Poll et al. 2017). LPS released into the circulation interact with lipoproteins, such as the High-Density Lipoproteins (HDL) and Low-Density Lipoproteins (LDL), but importantly with lipopolysaccharide-binding proteins (Vreugdenhil et al. 2001), which eventually promote LPS clearance through the hepatobiliary system (Pérez-Hernández et al. 2021). The LPS-binding protein and the bactericidal/permeability-increasing protein are closely related proteins that bind LPS, and present an important role in the host response to acute infections involving Gram-negative bacteria (Krasity et al. 2011). These proteins are members of the LTP/LPS-binding family of lipid binding and transfer proteins which also includes the cholesteryl-ester transfer protein (CETP), the phospholipid transfer protein (Beamer 2003), and now the cholesteryl-ester transfer protein isoform (CETPI), originally described by our research group years ago (Alonso et al. 2003; García-González et al. 2015).

CETPI was identified and found to be synthesized in the small intestine, and it is present in human plasma (Alonso et al. 2003). The only structural difference between CETP and CETPI resides in their C-terminal domain, where human CETPI presents a 41 C-terminal segment containing prolines, and a disordered conformation, instead of the normal 24 residues present as an α-helix in CETP (Alonso et al. 2003). Peptides derived from the C-terminal domain of CETPI bind LPS through electrostatic interactions, and the intravenous administration of one of these peptides, called VSAK, attenuates the harmful effects produced by LPS in a model of the systemic inflammatory response syndrome (Luna-Reyes et al. 2021). In addition, considering that the expression of CETPI in intestinal cells is upregulated in the presence of LPS (García-González et al. 2015), we aimed to evaluate the relation of the CETPI plasma levels with disease severity in Gram-negative sepsis. For this purpose, during the present study we collected plasma samples along a period of two years at the ICU of Instituto Nacional de Ciencias Médicas y Nutrición “Salvador Zubirán” (INCMNSZ), a tertiary care center located in Mexico City. In comparison to healthy subjects, we measured the plasma CETPI concentration in patients presenting infection, sepsis, and septic shock, and examined the correlation between CETPI with the plasma levels of LPS and a series of cytokines in all patients.

Our results suggest that an increased plasma level of CETPI in patients, associated to the presence of peptides derived from this protein, in correlation with their ability to bind and inactivate LPS, might be considered as a defense mechanism involved in the emergency host response to a Gram-negative infection, and therefore, also as indicators of disease severity.

## Materials and methods

### Study design

For this study, plasma samples from healthy subjects and patients with infection, sepsis, and septic shock with positive blood culture for Gram-negative bacteria were collected over a period of two years at the ICU of INCMNSZ (Mexico City). To quantify CETPI in plasma we employed an enzyme-linked immunosorbent assay (ELISA). The pro- and anti-inflammatory plasma cytokines were measured using the human cytokine magnetic bead panel Milliplex Map Kit. We determined the relationship between plasma levels of LPS and CETPI in patients with liver dysfunction. Also, the expression of CETPI in plasma was confirmed by Western Blot analysis, and the bands recognized by the anti-CETPI antibody identified by HPLC-mass spectrometry. Depletion of plasma albumin was used to determine the possible binding of peptides derived from the C-terminal domain of CETPI to this protein.

### Study population

For the development of this study at INCMNSZ, 47 healthy subjects, and 105 patients with infection, sepsis, and septic shock between 18 and 65 years of age were enrolled. All subjects provided written informed consent prior to the participation in the study. As controls, a total of 47 samples were obtained from healthy volunteer blood donors at the blood bank of INCMNSZ. The inclusion criteria were body mass index < 30 kg per m^2^, normal vital signs, oxygen saturation > 90%, with no evidence of infection or acute/chronic illnesses. Exclusion criteria included having consumed any drug seven days before sample collection, and the presence of an infection, inflammatory, and traumatic process in the six weeks before the acceptance for inclusion in the study.

Patients were divided into three groups: infection, sepsis, and septic shock. Baseline values for SOFA scores were determined upon patient arrival at the ICU, and the Delta SOFA score was calculated as the change in total SOFA score between baseline values and values obtained 24 h after ICU admission. The infection group included 50 subjects without evidence of organ failure defined by the Delta SOFA score lower than 2 points. The sepsis group comprised 28 sepsis patients with infection and organ failure defined as Delta SOFA score of 2 points or more. The septic shock group included 27 patients with sepsis and persisting hypotension requiring vasopressors to maintain a mean arterial pressure of 65 mmHg or greater and having a serum lactate level greater than 2 mmol/L (> 18 mg/dL) in the absence of hypovolemia. All patients had positive blood cultures for Gram-negative bacteria. Patients with trauma, renal replacement therapy, or patients that have received anti-inflammatory drugs 48 h prior to sample collection were excluded from the study.

Patients were clinically assessed by recording vital signs, mean arterial pressure, C-Reactive Protein (CRP), hemoglobin, albumin, procalcitonin (PCT), bilirubin, lactate, creatinine, HDL, leucocytes, platelets, and time of antibiotic treatment. Also, demographic data and baseline comorbidities (heart disease, lung disease, chronic liver dysfunction, immunosuppression, cancer, endocrinopathy, dyslipidemia, and autoimmune disease) were collected at the time of the patient’s admission. Patients were considered immunocompromised if they had severe neutropenia, and/or received steroid treatment or cytotoxic drugs. The source of infection was categorized into eight groups: abdomen, lung, urinary tract, soft tissue, sinusitis, empyema, central nervous system, and intravenous catheter. The criteria employed to define patients with liver dysfunction were: patients with known liver disease, basal SOFA score ≥ 1 in liver and coagulation item, or a previous diagnosis of cirrhosis.

This study was reviewed and approved by the Ethics Committee of the INCMNSZ (Reference number: 2252).

### Sample collection

All plasma samples were collected within 12 h of ICU admission, then samples were centrifuged for 10 min at 1500 rpm, and aliquots were immediately stored at − 80 °C. Blood cultures were taken in all patients upon arrival to the ICU. Only samples with positive blood culture for Gram-negative bacteria were included in the study.

### Western blot analysis

Protein from plasma samples was measured using the DC Protein Assay (Bio-Rad, Hercules, CA, USA). A total of 30 µg of protein were loaded and separated on 8% SDS-PAGE, and further transferred to PVDF membranes (Immobilon-P, Merck Millipore, Billerica, MA, USA). Membranes were blocked using 10% of Blotting-Grade Blocker (Bio-Rad), incubated with anti-CETPI (1:4000) overnight at 4 °C, and washed six times with TBST. Anti-CETPI antibodies from Alpha Diagnostic International Inc. (San Antonio, TX, USA), consist of antibodies raised using a synthetic peptide that corresponds to the last 12 amino acids of the C-terminal segment of CETPI. Membranes were incubated with a rabbit peroxidase-conjugated anti-chicken IgY secondary antibody (1:30,000) (Thermo Fisher Scientific, Waltham, MA, USA) for 60 min at room temperature (RT). Antibodies were diluted in a 5% of Blotting-Grade Blocker (Bio-Rad) solution. Protein bands were visualized using Immobilon^®^ Western from Merck Millipore on X-OMAT autoradiographic plates (Kodak, Rochester, NY, USA).

### Plasma cytokine measurements

For the measurement of cytokine levels in plasma, the human cytokine magnetic bead panel Milliplex^®^ Map Kit (Merck Millipore) was used to assess the levels of IL-1β, TNFα, IL-6, IL-8, IL-12 (p70), IFNγ, and IL-10. Plasma samples were analyzed in duplicate, and the procedure was carried out according to the manufacturer instructions. Briefly, 25 µL of control and patient plasmas were incubated with antibody-immobilized beads overnight at 4 °C. Bead-complexes, after being rinsed, were incubated with 25 µL of biotinylated detection antibody for 1 h with agitation. Next, 25 µL of Streptavidin–Phycoerythrin were added and incubated for 30 min at RT with agitation. After washing the plate, 150 µL of Drive Fluid were added to all wells. Plates were read on a MAGPIX employing the xPONENT software, and the Median Fluorescent Intensity analyzed.

### Plasma CETPI measured by ELISA

CETPI plasma levels were measured by an ELISA test employing 96-well Maxisorp plates (Thermo Fisher Scientific). 50 µL of dilutions of plasma samples were added into the appropriate wells and incubated overnight at 4 °C, then the plate washed out one time with Phosphate-Buffered Saline (PBS). The plate was blocked for 2 h using 2.5% Bovine Serum Albumin plus 2.5% of Blotting-Grade Blocker (Bio-Rad), and then washed out three times with PBS. Next, anti-CETPI (1:5000) antibodies were added into each well and the plate incubated for 90 min at 37 °C. The plate was washed out three times with PBS and further incubated with a rabbit peroxidase-conjugated anti-chicken IgY secondary antibody (Thermo Fisher Scientific) for 30 min at 37 °C. Finally, the plate was washed with PBS, TMB substrate was added to each well, and incubated for 15 min at RT. To stop the reaction, 50 µL of 2 M H_2_SO_4_ were added and the absorbance of samples measured at 450 nm using the Synergy HT microplate reader (BioTek Instruments, Inc., Winooski, VT, USA). CETPI plasma concentration of all samples was determined using a standard curve.

### Casein protease activity assay

Plasma protease activity was determined employing the Protease Activity Assay Kit (Abcam, Cambridge, UK), which uses FITC-Casein as a general protease substrate. Plasma samples were incubated with the protease substrate for 30 min at 25 °C, and fluorescence was measured at Ex/Em = 485/ 550 nm immediately after the protease substrate addition (R1) and at 30 min, after the incubation time (R2). Differences between both lectures (R2–R1), indicated the fluorescence of the unquenched FITC generated by the proteolytic digestion of the substrate. The plasma protease activity was reported as mU/mL. One unit was defined as the amount of protease that cleaves the substrate, to yield an amount of fluorescence equivalent to 1.0 µmol of unquenched FITC per minute at 25 °C.

### Plasma LPS quantification

Plasma LPS was measured using a competitive inhibition enzyme immunoassay (Cloud-Clone Corp, Houston, TX, USA). This assay employs a monoclonal antibody specific to lipopolysaccharide pre-coated onto a microplate. 50 µL of dilutions of standard, blank, and samples were added into the appropriate wells. A competitive inhibition reaction was launched between biotin-labeled lipopolysaccharide and the unlabeled lipopolysaccharide present in the samples with the pre-coated antibody. After incubation for 1 h at 37 °C, the unbound conjugate is washed off, and then Avidin conjugated to HRP added to each well. The plates were incubated for 30 min at 37 °C and washed five times. The substrate solution was added to each well and after the incubation time (30 min at 37 °C), 50 µL of stop reaction solution were added. The absorbance was measured at 450 nm using a Synergy HT microplate reader (BioTek Instruments, Inc.). The intensity of color developed represents the amount of bound HRP that is inversely proportional to the concentration of LPS in the sample.

### Albumin depletion

Albumin depletion of plasma samples was performed using the Pierce Albumin Depletion Kit (Thermo Fisher Scientific). The kit consists of a high-capacity, immobilized Cibacron Blue dye agarose resin, which binds the albumin present in the plasma. Briefly, the resin was transferred into a spin column to be washed employing the Binding/Wash Buffer. After, the plasma sample was added and incubated for 1–2 min at RT. The spin column was centrifuged, and the flow-through was re-applied to the spin column and incubated for another 1–2 min at RT to ensure maximal albumin binding. After centrifugation, we stored the flow-through and placed the spin column in a new collection tube. The column was washed to release unbound proteins and albumin eluted with a solution of 20 mM sodium phosphate, 250 mM sodium thiocyanate, pH 7.2. The protein content was determined in the collected fractions and were further analyzed by SDS-PAGE.

### Statistical analysis

Data are expressed as frequencies for categorical variables and as a mean with standard deviation (SD) or median with interquartile range (IQR) for continuous variables according to their distribution as assessed by Shapiro–Wilk normality test. When variables were not normally distributed, differences between groups were analyzed employing the Mann Whitney U-test, or the Kruskal–Wallis test with the Dunn's Multiple Comparison Test. In the case of variables with a normal distribution, the statistical analysis was performed using Student’s t-tests.

The plasma values of CETPI and cytokines were transformed to logarithm (log 10) before performing the correlations analysis to normalize the distribution of data. Correlations between cytokines and CETPI plasma levels were assessed by the Pearson’s correlation coefficient. Statistical test, significance level, n-numbers for each analysis are stated in figure legends. Differences were considered statistically significant at p < 0.05. The statistical analyses were performed employing the GraphPad Prism software (GraphPad Software, La Jolla, CA), and JMP 16.1.0 (SAS Institute, Cary NC, USA).

## Results

### Subjects and clinical parameters

Blood samples from 47 healthy subjects attending the blood bank at INCMNSZ for blood donation, were collected as controls, this group included 22 males and 25 females. Also, a total of 105 ICU patients were enrolled in this study; 50 patients with infection (Delta SOFA score < 2), and 55 patients with sepsis and septic shock (Delta SOFA score > 2), all patients with positive blood culture for Gram-negative bacteria (Table 1). The mean age in the overall population was 50.00 years (37.50–57.50), of which 55 (52.38%) were female and 50 (47.62%) male.

**Table 1:** Demographic, clinical and laboratory parameters of patients

	Total (n = 105)	Patients with Delta SOFA score < 2 (n = 50)	Patients with Delta SOFA score > 2 (n = 55)	*p* value
Gender, male	50 (47.62%)	27 (54.00%)	23 (41.82%)	0.24
Gender, female	55 (52.38%)	23 (46.00%)	32 (58.18%)	
Age (years)	50.00 (37.50–57.50)	54.50 (39.00–58.00)	47.00 (35.00–55.00)	0.17
Etiologic agent (%)				
* Escherichia coli*	61 (58.10%)	28 (56.00%)	33 (60.00%)	0.36
* Klebsiella pneumoniae*	12 (11.43%)	7 (14.00%)	5 (9.09%)	
* Pseudomonas aeruginosa*	12 (11.43%)	8 (16.00%)	4 (7.27%)	
Site of infection (%)				
Abdominal	71 (67.62%)	32 (64.00%)	39 (70.91%)	0.15
Urinary tract	20 (19.05%)	12 (24.00%)	8 (14.55%)	
Intravenous catheter	5 (4.76%)	3 (6.00%)	2 (3.64%)	
Pulmonary	4 (3.81%)	0 (0.00%)	4 (7.27%)	
Comorbidities				
Dyslipidemia	61 (58.10%)	32 (64.00%)	29 (52.73%)	0.89
Immunosuppression	55 (52.38%)	26 (52.00%)	29 (52.73%)	
Cancer	55 (52.38%)	28 (56.00%)	27 (49.09%)	
Liver dysfunction	27 (25.71%)	12 (24.00%)	15 (27.27%)	
Laboratory tests				
CRP (mg/L)	14.00 (7.30–19.80)	10.75 (4.80–16.87)	15.20 (9.47–21.50)	< 0.05
PCT (ng/mL)	7.40 (1.80–17.90)	3.10 (1.33–8.58)	11.40 (3.90–24.33)	< 0.01
Hemoglobin (g/dL)	10.27 ± 2.63	10.38 ± 2.62	10.17 ± 2.66	0.47
Serum Albumin (g/dL)	3.17 ± 0.73	3.26 ± 0.70	3.09 ± 0.76	0.23
Total bilirubin (mg/dL)	1.40 (0.62–4.90)	0.90 (0.60–2.93)	2.20 (0.90–7.40)	< 0.01
Lactate (mmol/L)	2.40 (1.40–4.00)	1.65 (1.18–2.60)	3.45 (1.63–4.20)	< 0.01
Creatinine (mg/dL)	0.90 (0.65–1.40)	0.80 (0.50–1.06)	1.00 (0.70–1.90)	< 0.01
White blood cells (× 10^9^/L)	6.95 (0.35–12.30)	7.25 (0.60–12.18)	6.65 (0.10–12.30)	0.87
Platelet (× 10^3^/µL)	107.0 (21.00–243.00)	162.50 (26.25–265.00)	100.00 (16.00–216.00)	0.19
HDL (mg/dL)	35.19 ± 17.71	36.88 ± 17.57	33.65 ± 17.89	0.40
Total cholesterol (mg/dL)	138.50 (113.30–181.80)	139.50 (109.00–201.30)	136.00 (115.80–165.50)	0.56
LDL (mg/dL)	75.00 (48.25–109.30)	77.00 (48.75–118.30)	73.00 (47.50–101.50)	0.46
Delta SOFA score	0–11	0–1	2–11	

Data are reported as mean (± SD), median (IQR), and n (%). Comparisons were performed with Fisher’s exact test or Pearson’s Chi-squared test, and either Mann–Whitney U test or Student’s t-test. C-reactive protein (CRP); procalcitonin (PCT); High-Density Lipoprotein (HDL); Low-Density Lipoprotein (LDL); sequential organ failure assessment (SOFA)

In all patients, the most prevalent bacteria isolated from the bloodstream was *Escherichia coli* (58.10%), and the main sites of infection were the abdomen (67.62%), and the urinary tract (19.05%) (Table 1). In patients with Delta SOFA score < 2, the main comorbidities corresponded to dyslipidemia (64.00%), cancer (56.00%), immunosuppression (52.00%), and liver dysfunction (24.00%). In patients with Delta SOFA score > 2, dyslipidemia (52.73%), immunosuppression (52.73%), cancer (49.09%), and liver dysfunction (27.27%) were also the principal comorbidities (Table 1).

The main clinical parameters of patients are shown in Table 1, CRP levels were lower in patients with Delta SOFA score < 2 (10.75 [4.80–16.87]), than in patients with Delta SOFA score > 2 (15.20 [9.47–21.50], p < 0.05). PCT levels were also higher in patients with Delta SOFA score > 2 (11.40 [3.90–24.33]) compared to patients with Delta SOFA score < 2 (3.10 [1.33–8.58], p < 0.01). Also, significant differences were observed in the levels of total bilirubin, lactate, and creatinine between patients with Delta SOFA score > 2, and those with Delta SOFA score < 2 (Table 1).

### The concentration of CETPI in plasma is higher in patients with Gram-negative bacteraemia

**Fig. 1:**
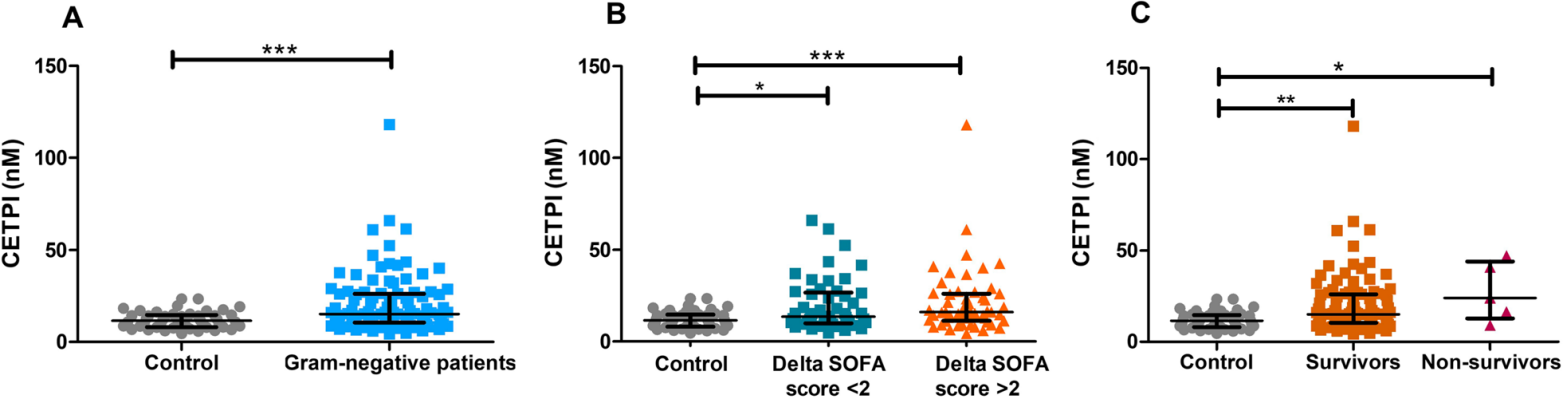
CETPI plasma levels in patients with Gram-negative bacteraemia. **A** CETPI plasma concentration of patients with a positive blood culture for Gram-negative bacteria (n = 105) and control subjects (n = 47). **B** CETPI plasma concentration of patients with Delta SOFA score ˂2 (n = 50), or ˃ 2 (n = 55) and control subjects (n = 47). **C** CETPI plasma concentration between survivors (n = 100), non-survivors (n = 5), and control subjects (n = 47). Data are shown as median with IQR. *p < 0.05, **p < 0.01, ***p < 0.001. Differences between groups were assessed using Mann- Whitney test or Kruskal–Wallis test and subsequent Dunn’s Multiple Comparison test. Samples were assessed in duplicate in ELISA assays

To explore the relation between CETPI and disease severity in infections due to Gram-negative bacteria, circulating levels of CETPI were measured in the plasma from controls and patients with infection (Delta SOFA score < 2), sepsis, and septic shock (Delta SOFA score > 2) employing ELISA. We found that patients with an infectious process due to Gram-negative bacteria had elevated circulating levels of CETPI when compared to control subjects (15.21 nM [10.52–26.23] vs 11.58 nM [8.05–14.71], p < 0.001) (Fig. 1A), showing a clear difference in the distribution of the data between controls and patients above 18 nM (Additional file 1: Fig. S1). Although differences in CETPI plasma levels were found between control subjects and patients with Delta SOFA score < 2 (11.58 nM [8.05–14.71] vs 13.54 nM [9.99–26.65], p < 0.05), and with those with Delta SOFA score > 2 (11.58 nM [8.05–14.71] vs 16.00 nM [11.30–26.11], p < 0.001), no differences were observed between patients grouped according with the Delta SOFA score greater or less than 2 (Fig. 1B). Patients who had a severe outcome (in-hospital death), did not have significantly differences in CETPI levels than those with a non-severe outcome (Fig. 1C).

### CETPI and plasma cytokines correlate with SOFA score in patients with Gram-negative bacteraemia

**Fig. 2:**
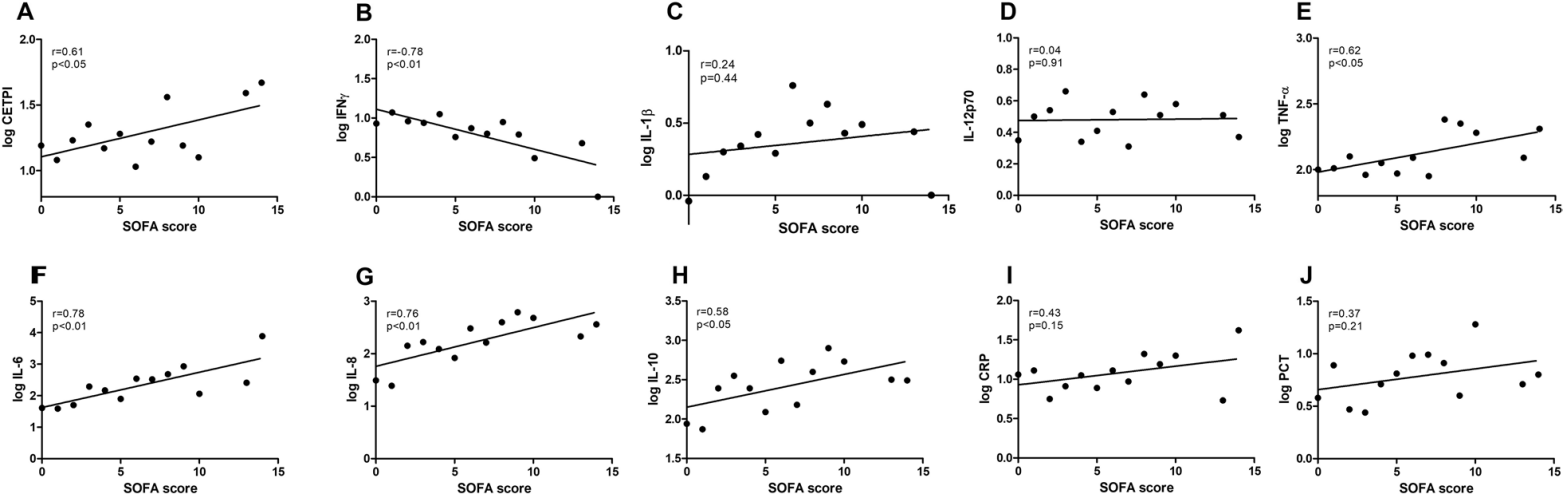
Correlation between SOFA score measured within 12 h of ICU admission  with CETPI and cytokines in patients with Gram-negative bacteraemia. Pearson correlations show associations between SOFA score with the mean values of CETPI and relevant cytokines in patients. **A** CETPI. **B** IFNγ. **C** IL-1β. **D** IL-12p70. **E** TNF-α. **F** IL-6. **G** IL-8. **H** IL-10. **I** CRP. **J** PCT. The levels of the 7 cytokines and CETPI, CRP, PCT were transformed to common logarithm values for the correlations. SOFA: Sequential Organ Failure Assessment; CRP: C-Reactive Protein; PCT: Procalcitonin

In parallel to the determination of plasma levels of CETPI, plasma cytokine concentrations of IL-1β, TNFα, IL-6, IL-8, IL-12 (p70), IFNγ, and IL-10 were also measured (Fig. 2). Given that higher CETPI levels were found in patients compared with healthy subjects, and considering its ability to bind LPS, a correlation between SOFA scores (measured within 12 h of ICU admission) with CETPI, and plasma cytokines were carried out. We found that SOFA scores positively correlate with CETPI (r = 0.61, p < 0.05), TNF-α (r = 0.62, p < 0.05), IL-6 (r = 0.78, p < 0.01), IL-8 (r = 0.76, p < 0.01), and IL-10 (r = 0.58, p < 0.05); whereas a negative correlation was found with IFNγ (r = − 0.78, p < 0.01) (Fig. 2A–J).

### CETPI plasma levels negatively correlate with IL-1β, IL-8, and IL-10 in septic shock patients

**Fig. 3:**
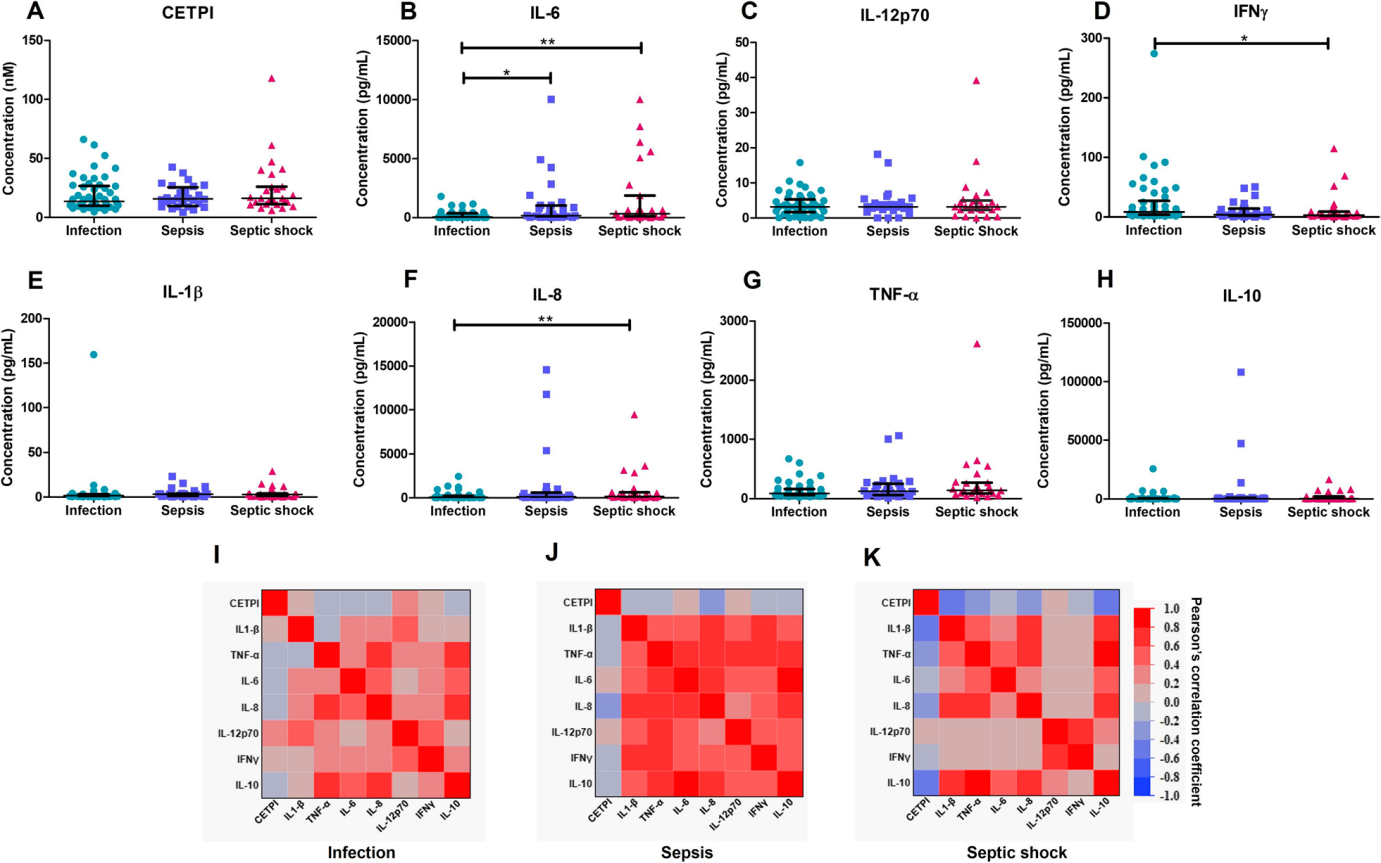
CETPI and cytokines plasma levels in patients with infection, sepsis, and septic shock due to Gram-negative bacteria. **A** CETPI. **B** Interleukin-6 (IL-6). **C** Interleukin-12p70 (IL-12p70). **D** Interferon γ (IFNγ). **E** Interleukin-1 beta (IL-1β). **F** Interleukin-8 (IL-8). **G** Tumor necrosis factor-alpha (TNF-α). **H** Interleukin-10 (IL-10). Data are shown as median with IQR; *p < 0.05, **p < 0.01; Differences between groups were assessed using Kruskal–Wallis test and subsequent Dunn’s Multiple Comparison test. **I** Pearson’s correlation values between the 7 cytokines and CETPI in patients with infection, **J** sepsis, and **K** septic shock. The red color indicates a positive correlation, and the blue color a negative correlation

To explore the association between CETPI plasma levels with cytokines implicated in the pathophysiology of Gram-negative infections, a correlation was carried out. First, we analyzed CETPI and cytokine expression in patients with infection, sepsis, and septic shock (Fig. 3A–H). Although no differences in CETPI levels were found in the three patient groups, in septic shock patients CETPI levels tend to increase (Fig. 3A) (Additional file 1: Fig. S1); also, significant differences in the levels of IL-6, IFNγ, and IL-8 were found. IL-6 levels were found to be fivefold higher in septic shock patients, and 2.7-fold higher in sepsis patients, compared with patients with infection (319.00 pg/mL [105.1–1870.00] and 166.70 pg/mL [56.98–1033.00] vs 61.81 pg/mL [21.56–370.5]; p < 0.01 and p < 0.05) (Fig. 3B). IFNγ levels were 2.7-fold lower in septic shock patients compared with patients with infection (3.2 pg/mL [2.23–9.21] vs 8.58 pg/mL [3.84–27.01], p < 0.05) (Fig. 3D). IL-8 levels were 3.2-fold higher in septic shock patients compared with patients with infection (182.60 pg/mL [97.34–621.70] vs 55.40 pg/mL [21.74–218.30], p < 0.01) (Fig. 3F).

We next assessed the correlation between the full set of cytokines and CETPI in patients with infection, sepsis, and septic shock through the Pearson correlation coefficient. There were positive correlations between the pro-inflammatory cytokines in infection patients, and more importantly, in sepsis patients (Fig. 3I, J) (Additional file 1: Table S1 and S2). However, in septic shock patients, the correlation between IFNγ and IL-12 (p70) with CETPI, IL-1β, TNF-α, IL-6, IL-8, and IL-10, were not significant (Fig. 3K). IL-10 showed positive correlations with the other six cytokines studied in patients with sepsis (Fig. 3J), and with IL-1β, TNF-α, IL-6, and IL-8 in patients with septic shock (Fig. 3K) (Additional file 1: Table S2 and S3). Interestingly, there were negative correlations between CETPI with IL-1β, IL-8, and IL-10 in septic shock patients (Fig. 3K) (Additional file 1: Table S3).

### Patients with liver dysfunction present increased plasma concentrations of LPS and CETPI

**Fig. 4:**
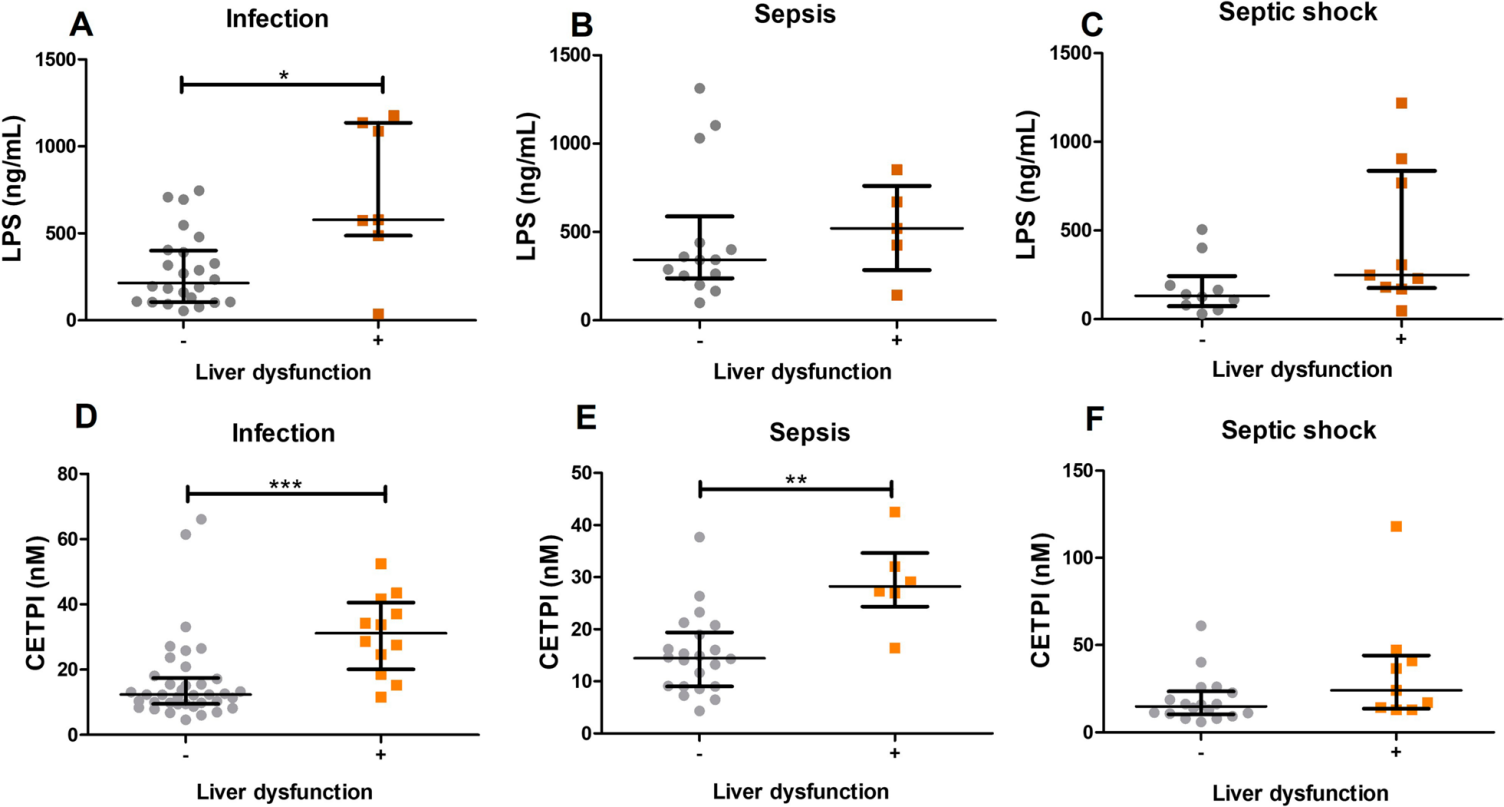
LPS and CETPI plasma levels in patients with or without liver dysfunction. LPS plasma levels from patients with **A** Infection (n = 31); **B** Sepsis (n = 19); **C** Septic shock (n = 19), classified according to the presence or absence of liver dysfunction. CETPI plasma levels from patients with **D** Infection (n = 50); **E** Sepsis (n = 28); **F** Septic shock (n = 27), classified according to the presence or absence of liver dysfunction. Differences between groups were assessed using Mann–Whitney test. Data are shown as median with IQR. *p < 0.05, **p < 0.01, ***p < 0.001

Considering that the main site of infection in all patients studied corresponds to an intra-abdominal origin (Table 1), and that hepatic cells are involved in the clearance of LPS of intestinal origin, we analyzed the levels of LPS and CETPI in the plasma of patients with and without liver dysfunction (Fig. 4). Plasma LPS levels were higher in patients that presented liver dysfunction (Fig. 4A–C), with differences in patients with infection versus patients with infection but without liver dysfunction (577.90 pg/mL [487.20–1136.00] vs 214.90 pg/mL [105.90–400.70], p < 0.05) (Fig. 4A). Interestingly, the plasma concentration of CETPI was also found to be higher in infection (31.19.63 nM [20.07–40.58]), and sepsis patients (28.21 nM [24.34–34.65]) with liver dysfunction compared with patients with infection (12.35 nM [9.50–17.41]) and sepsis (14.45 nM [9.04–19.39]) without this comorbidity (Fig. 4D, E). Although the differences showed not to be statistically significant, a similar increase in CETPI concentration was observed in septic shock patients (24.10 nM [13.54–44.07]) with respect to patients without liver dysfunction (14.84 nM [10.29–23.44]) (Fig. 4F). Besides, it is shown that a high plasma CETPI and LPS levels correlate in patients with infection and sepsis due to Gram-negative bacteria (Additional file 1: Fig. S2).

### Proteolytic activity upon CETPI is associated with disease severity

**Fig. 5:**
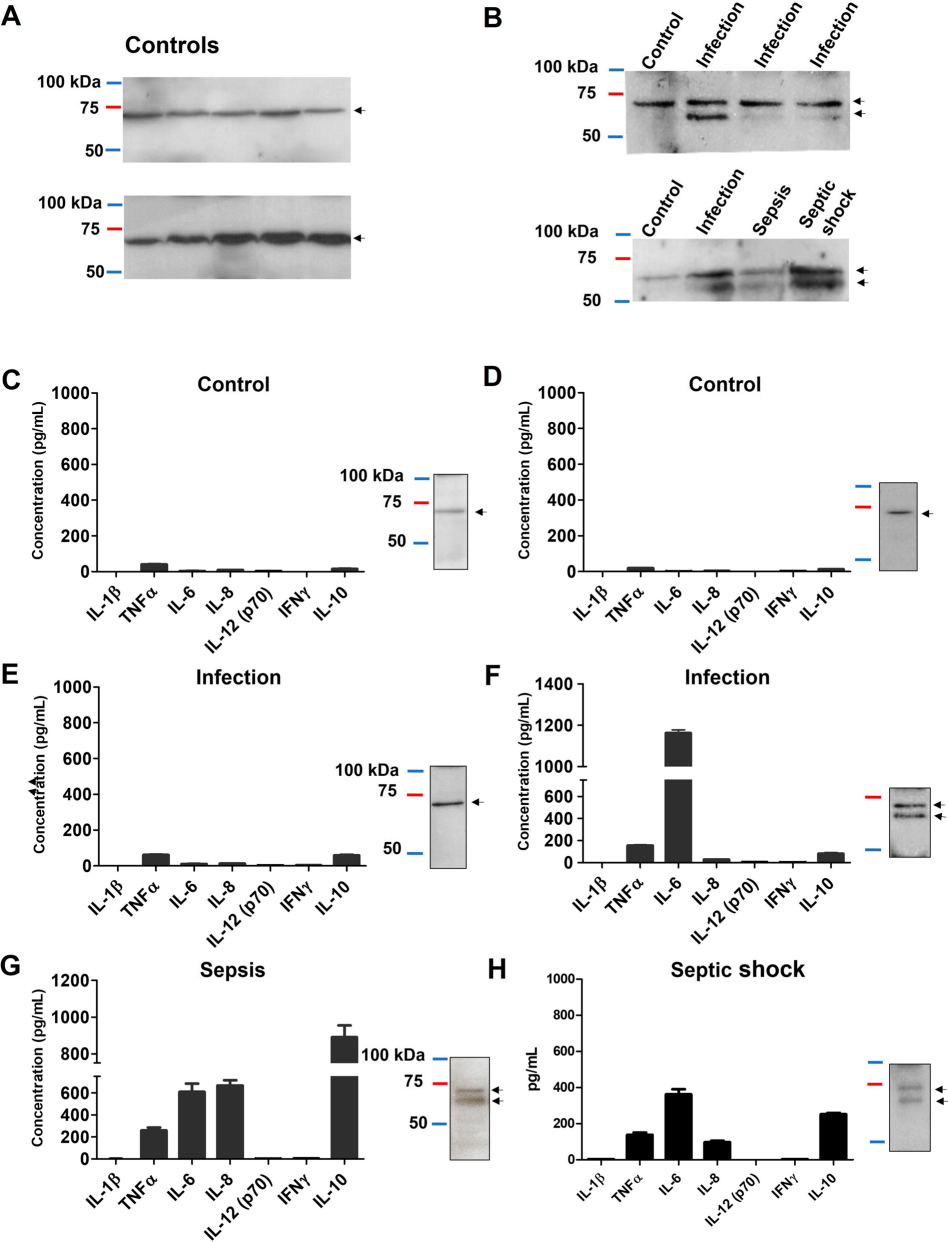
Association between cytokine plasma levels, and the presence of CETPI in the plasma of representative samples from control subjects and patients. **A** CETPI expression in plasma samples from control subjects. **B** Top: CETPI expression in plasma samples from control subjects and patients with infection. Down: CETPI expression in plasma samples from control subjects and patients with infection, sepsis, and septic shock. **C**, **D** Plasma samples obtained from control subjects; **E**, **F** patients with infection; **G** sepsis; and **H** septic shock. Left panels show the level of IL-1β, TNFα, IL-6, IL-8, IL-12 (p70), IFNγ, and IL-10 (Data are presented as mean ± SEM). At the right of each graph, the protein band that corresponds to CETPI (arrow tips) determined by western blot analysis, is shown. The same plasma samples were used for both measurements of plasma cytokines and CETPI

We analyzed the CETPI expression employing a Western Blot analysis in plasma samples of both controls and patients (Fig. 5). In the plasma of controls, it was detected the expected band of 69 kDa (Fig. 5A), whereas, when plasma samples from patients were analyzed, together with the regular protein band corresponding to CETPI (69 kDa), a lower molecular weight protein band (~ 67 kDa) appeared (Fig. 5B). Although a basal level of CETPI was detected in control plasma samples, it did not show any type of correlation with the negligible concentration of inflammatory cytokines (Fig. 5C, D). However, when plasma samples from patients with infection were analyzed, the presence of the 67 kDa band was associated with the increased level of inflammatory cytokines, especially with IL-6 (Fig. 5E, F) (Additional file 1: Table S4). Moreover, when plasma samples from patients with sepsis and septic shock were analyzed, this phenomenon was exacerbated showing a clear association with the presence of the ~ 67 kDa accessory protein band, and a marked increase in IL-6 and IL-10 (Fig. 5G, H) (Additional file 1: Table S4).

**Fig. 6:**
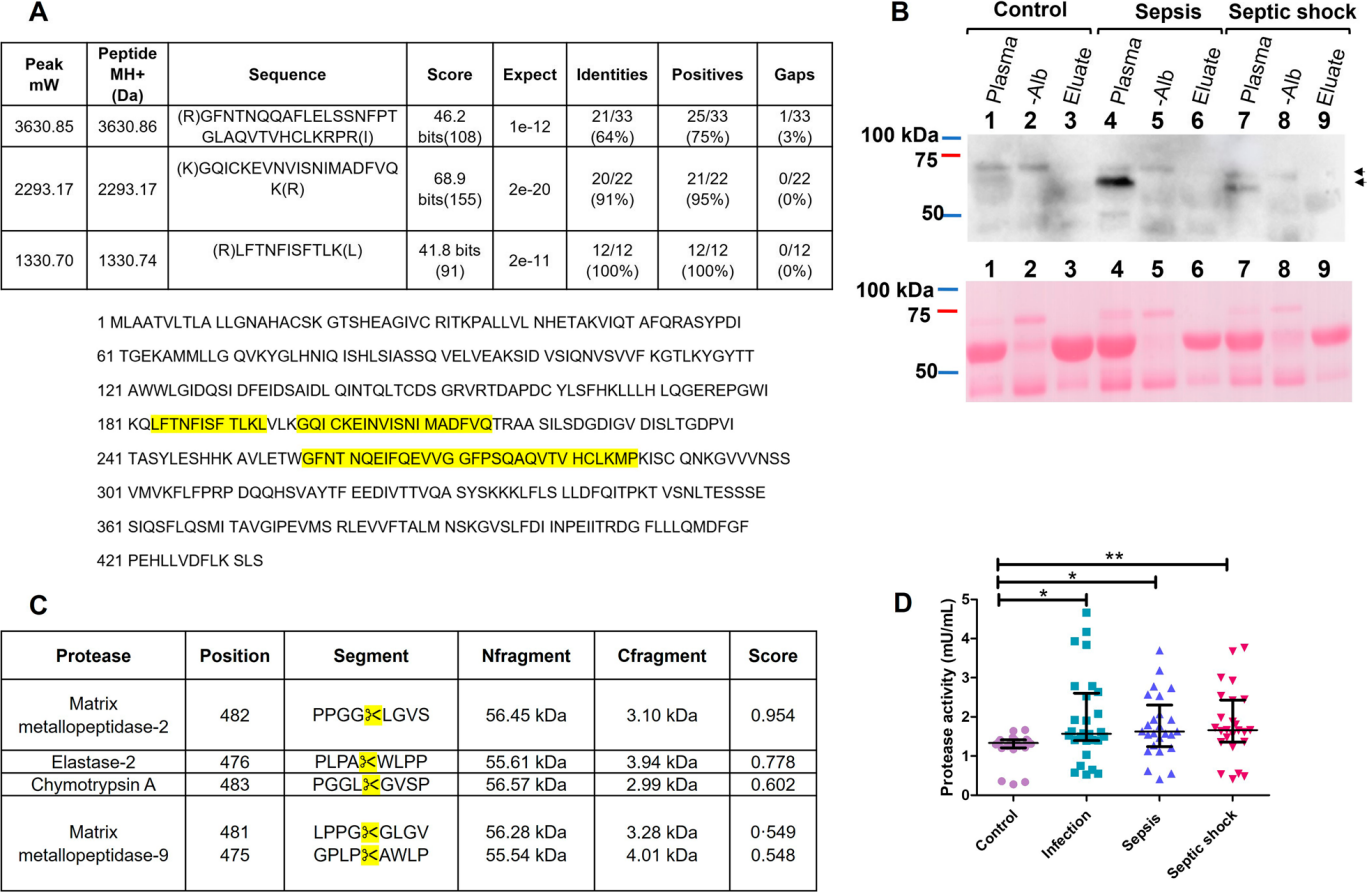
Analysis of the 69 kDa and 67 kDa bands identified by western blotting that correspond to CETPI and peptides derived from its C-terminal sequence. **A** Alignment parameters of peptides identified by HPLC-mass spectrometry contained in the 69 kDa CETPI band employing Homo sapiens CETP sequences (GenBank: AAV38867.1). Peptides identified by HPLC-mass spectrometry are highlighted in the CETP sequence. **B** Top: Immunodetection of CETPI in plasmas isolated from control subjects and patients before and after albumin depletion. Down: Same membrane stained with Ponceau S. Control plasma samples (lanes 1–3). Plasma samples from sepsis patient (lanes 4–6). Plasma samples from septic shock patient (lanes 7–9). Albumin-depleted plasma samples (lanes 2, 5, 8). Albumin fraction recovered after elution of plasmas through the column (lanes 3, 6, 9). **C** Protease activity prediction upon the C-terminal domain of CETPI employing Procleave (Li et al. 2020), ✂ indicates predicted cleaved sites. **D** Protease activity of plasmas obtained from control subjects (n = 21), infection (n = 28), sepsis (n = 25), and septic shock patients (n = 25). Data are shown as median with IQR. *p < 0.05, **p < 0.01

In order to find out the origin of the ~ 67 kDa band observed in the Western blot analysis, we employed HPLC-mass spectrometry. Interestingly, sequences directly related to the sequence for CETPI were found (Fig. 6A). However, the question arises, how an anti-CETPI antibody raised against the C-terminal sequence of CETPI, not present in CETP, permitted us to identify a second band in the western blot analysis? We found that the largest percentage of peptides identified by HPLC-mass spectrometry analysis of the ~ 67 kDa band, corresponds to sequences belonging to albumin, a well-known protein that readily binds peptides. Therefore, we decided to find out if contained in the plasma of sepsis and septic shock patients, albumin that normally shows an average molecular weight of 66.5 kDa, might contain bound-peptides derived from the C-terminal segment of CETPI. For this purpose, following a procedure to eliminate albumin from the plasma of sepsis and septic shock patients, samples were eluted through columns containing an albumin-binding resin. Interestingly, we do not detect the ~ 67 kDa in the albumin-depleted plasma samples (Fig. 6B). Considering that the elution of the albumin of the columns was done employing harsh conditions, peptides initially bound to albumin most probably became detached and eliminated, and therefore not detected in association with albumin (Fig. 6B). This set of experiments, confirm that peptides derived from the C-terminal region of CETPI, when bound to albumin are responsible for the positive Western blot signal found at ~ 67 kDa (Fig. 6B).

Overall, these results suggest the possibility that the activation of proteolytic enzymes might occur in the plasma of patients with sepsis and septic shock, therefore, the proteolytic activity upon CETPI could be generating peptides that eventually end up binding to albumin. In order to further explore this possibility, an analysis of the most susceptible sites for proteolytic cleavage at the C-terminal domain of CETPI was carried out. Interestingly, we found such positions where enzymes matrix-metallopeptidase-2 (MMP-2), elastase-2, chymotrypsin A, and matrix-metallopeptidase-9 (MMP-9) might act (Fig. 6C). This analysis and the increased protease activity found in the plasma samples from all patient groups, in comparison with controls (Fig. 6D), support the hypothesis that the 67 kDa accessory protein band, corresponding to albumin, contains cleaved peptides derived from the C-terminal segment of CETPI, and therefore identified by the anti-CETPI antibody.

## Discussion

Sepsis as a life-threatening syndrome with important variations in terms of incidence and mortality across the world, affects according to the World Health Organization, around 50 million people every year associated with 11 million deaths (WHO 2020). Therefore, this syndrome is responsible for approximately 20% of total deaths in the world in a single year (Rudd et al. 2020). Several pathophysiological alterations are present in sepsis, where a balanced response to the infection is essential to improve the survival rate. In this study, we describe the existing correlation between the severity of the disease, CETPI and LPS plasma levels during Gram-negative bacteraemia.

Our results show the presence of a high plasma CETPI concentration in the patients with an infectious process due to Gram-negative bacteria, in comparison to the control group. Since we have previously reported the presence of CETPI in the plasma of healthy subjects (Alonso et al. 2003), here as increased concentrations of CETPI have been found in patients with Gram-negative bacteraemia, we consider its role as a potential LPS-binding protein, and therefore as a participant in the physiological response to LPS. Although no direct differences were found in the concentration of CETPI between patients, an important correlation was found between CETPI concentrations and the SOFA score, indicating an association with the degree of organ dysfunction/failure (Lambden et al. 2019). A direct correlation between the SOFA score and IL-6, TNF-α, IL-8, and IL-10 was also observed. Interestingly, TNF-α and IL-6 as cytokines involved in endothelial damage, and multiple organ dysfunction syndrome, are often used as biomarkers for sepsis (Miguel-Bayarri et al. 2012; Molano Franco et al. 2019; Grondman et al. 2020). Whereas, IL-8 has been proposed as a prognostic factor in septic patients (Livaditi et al. 2006; Anderson et al. 2019),  and IL-10 has been used as an indicator of a hypoinflammatory phenotype (Barichello et al. 2022). On the other hand, IFNγ that negatively correlates with the SOFA score, is known to be required during the host defense against pathogens, therefore contributing to a worse prognosis if the infection is not being controlled (Ono et al. 2018). Patients with septic shock present higher IL-6 and IL-8 plasma levels and lower levels of IFNγ than patients with infection, showing that an impaired immune response contributes to a worse prognosis. Our results also show a positive correlation between IL-10 and pro-inflammatory cytokines, whose simultaneous presence has been associated with the pathogenesis of sepsis (Matsumoto et al. 2018).

Experiments from our group where LPS-treated rabbits were administered with a peptide derived from the carboxy-end segment of CETPI, showed attenuated circulating levels of pro-inflammatory molecules (Luna-Reyes et al. 2021). During the present investigation, based on the high levels of CETPI found in patients with Gram-negative bacteraemia, we explored its relationship with the cytokine plasma levels, and found negative correlations between CETPI with IL-1β, IL-8, and IL-10. These results are consistent with the LPS-binding role of CETPI, showing an interesting correlation with the release of IL-8 by the gut (García-González, et al. 2015; Luna-Reyes et al. 2021; Liu et al. 2006; Angrisano et al. 2010).

An in vitro study carried out by our group several years ago, shows a clear correlation between an increase in CETPI expression by Caco-2 cells and small intestine cells, under LPS stimulation (García-González, et al. 2015). Considering that CETPI apparently is only synthesized by intestinal cells, the increased level of LPS in circulation could promote the overexpression of CETPI in the intestinal epithelium as an early protective mechanism against the harmful action of LPS. Since LPS either entering the circulatory system from the intestinal tract through the portal circulation, or due to the antibiotic action upon bacteria, find their way to the liver, a key organ in the deactivation and clearance of LPS (Pérez-Hernández et al. 2021; Shao et al. 2007), our results are consistent with the fact that patients presenting liver dysfunction show an increased LPS plasma level (Bode et al. 1987; Parlesak et al. 2000; Thuy et al. 2008; Nier et al. 2020). This finding associated with the fact that patients showing liver dysfunction present even higher plasma CETPI levels, place CETPI as a promising biomarker of disease severity.

We consider this a clear response to infection and endotoxemia caused by the presence of LPS in the bloodstream following antimicrobial treatment, and/or when the intestinal epithelium starts to be compromised. Although endotoxemia might have other origins such as trauma, if we consider the gut as a major source of infection, the alteration of the intestinal barrier might be considered an important cause for this clinical condition (Pérez-Hernández et al. 2021).

The overexpression of CETPI associated with the formation of peptides secondary to proteolytic cleavage, seems to represent what it can be considered, a true emergency response system, switched-on at the start of an infection, and further developed in sepsis and septic shock. As consistently shown here with our western blot analysis of plasmas from all patient groups, the correlation between the appearance of the ~ 67 kDa electrophoretic band associated with the presence of proteolysis and generation of CETPI derived peptides, higher IL-6 levels, and organic failure, can be concurrent with other indicators of disease severity (Schulte et al. 2013; Fan et al. 2016).

Since the presence of systemic proteolysis has been associated with a high mortality rate in septic shock patients (Bauzá-Martinez et al. 2018), the fact that we did not find significant differences between infection, sepsis, and septic shock patients, seems to be related to the low mortality rate (4.8%) observed by us. Taking into account that CETPI is expressed in small intestine cells (Alonso et al. 2003), associated with the fact that the main source of infection in our patients is abdominal, proteolysis of CETPI by matrix metalloproteinases (MMPs) and serine proteases cannot be ruled out. In support of this possibility, the analysis for protease prediction performed by us shows that the C-terminal domain of CETPI presents potential cleavage sites for chymotrypsin A, elastase-2, MMP-9, and MMP-2.

Considering that CETPI might correspond to a protein that binds LPS in vivo, one of the limitations of this study is that we only included patients with positive blood cultures for Gram-negative bacteria. Therefore, it will be also of interest to explore the physiological role of CETPI in patients presenting other etiologies  and a non-infectious systemic inflammatory response. Also, data presented here, corresponds to clinical data and plasma samples obtained within 12 h of ICU admission, thus, both the clinical information and the analysis of plasmas collected 48 h after admission, are currently being studied and should be reported soon.

## Conclusion

This study provides new insights into the role of CETPI in sepsis due to Gram-negative bacteria. Given that a fine balance control given by diverse immune responses is essential in the prognosis of a septic patient, CETPI can be considered as a new LPS-binding protein, and therefore as a novel player in the pathophysiology of sepsis.

## Supplementary Information

**Additional file 1: Figure S1.** Empirical cumulative distribution of the CETPI measurements. Empirical cumulative distribution of CETPI plasma concentration of controls (n = 47) and patients with infection (n = 50), sepsis (n = 28), and septic shock (n = 27), with a positive blood culture for Gram-negative bacteria. **Figure S2**. LPS correlates with CETPI plasma levels in patients with Gram-negative bacteraemia. Correlation between LPS with CETPI levels in patients with A infection (n = 31), B sepsis (n = 19), and C septic shock (n = 19). Spearman correlations show associations between LPS and CETPI in patients with infection and sepsis. **Table S1.** Probability of correlation between cytokines and CETPI in patients with infection shown in Fig. 3I. **Table S2.** Probability of correlation between cytokines and CETPI in patients with sepsis shown in Fig. 3 J. **Table S3.** Probability of correlation between cytokines and CETPI in patients with septic shock shown in Fig. 3K. **Table S4**. Clinical parameters of patients with E, F infection, G sepsis, and H septic shock shown in Fig. 5.

## Data Availability

All data generated or analyzed during this study are included in the paper and its supplementary information files. Also, relevant data are available from the authors upon reasonable request.

## Abbreviations

CETPI: Isoform of the cholesteryl-ester transfer protein
LPS: Lipopolysaccharides
IL-1β: Interleukin-1 beta
TNFα: Tumor necrosis factor-alpha
IL-6: Interleukin-6
IL-8: Interleukin-8
IL-12p70: Interleukin-12p70
IFNγ: Interferon γ
IL-10: Interleukin-10
SOFA: Sequential Organ Failure Assessment
ICU: Intensive care unit
HDL: High-density lipoproteins
LDL: Low-density lipoproteins
CETP: Cholesteryl-ester transfer protein
ELISA: Enzyme-linked immunosorbent assay
INCMNSZ: Instituto Nacional de Ciencias Médicas y Nutrición “Salvador Zubirán”
CRP: C-reactive protein
PCT: Procalcitonin
RT: Room temperature
PBS: Phosphate-Buffered Saline
SD: Standard deviation
IQR: Interquartile range
MMP-2: Matrix-metallopeptidase-2
MMP-9: Matrix-metallopeptidase-9
MMPs: Matrix metalloproteinases

## References

[CR1] Alonso AL, Zentella-Dehesa A, Mas-Oliva J (2003). Characterization of a naturally occurring new version of the cholesterol ester transfer protein (CETP) from small intestine. Mol Cell Biochem.

[CR2] Anderson BJ, Calfee CS, Liu KD, Reilly JP, Kangelaris KN, Shashaty MGS (2019). Plasma sTNFR1 and IL8 for prognostic enrichment in sepsis trials: a prospective cohort study. Crit Care.

[CR3] Angrisano T, Pero R, Peluso S, Keller S, Sacchetti S, Bruni CB (2010). LPS-induced IL-8 activation in human intestinal epithelial cells is accompanied by specific histone H3 acetylation and methylation changes. BMC Microbiol.

[CR4] Barichello T, Generoso JS, Singer M, Dal-Pizzol F (2022). Biomarkers for sepsis: more than just fever and leukocytosis-a narrative review. Crit Care.

[CR5] Bauzá-Martínez J, Aletti F, Pinto BB, Ribas V, Odena MA, Díaz R (2018). Proteolysis in septic shock patients: plasma peptidomic patterns are associated with mortality. Br J Anaesth.

[CR6] Beamer LJ (2003). Structure of human BPI (bactericidal/permeability-increasing protein) and implications for related proteins. Biochem Soc Trans.

[CR7] Bode C, Kugler V, Bode JC (1987). Endotoxemia in patients with alcoholic and non-alcoholic cirrhosis and in subjects with no evidence of chronic liver disease following acute alcohol excess. J Hepatol.

[CR8] Cecconi M, Evans L, Levy M, Rhodes A (2018). Sepsis and septic shock. The Lancet.

[CR9] Fan SL, Miller NS, Lee J, Remick DG (2016). Diagnosing sepsis—the role of laboratory medicine. Clin Chim Acta.

[CR10] Font MD, Thyagarajan B, Khanna AK (2020). Sepsis and Septic Shock—basics of diagnosis, pathophysiology and clinical decision making. Med Clin North Am.

[CR11] García-González V, Gutiérrez-Quintanar N, Mas-Oliva J (2015). The C-terminal domain supports a novel function for CETPI as a new plasma lipopolysaccharide-binding protein. Sci Rep.

[CR12] Grondman I, Pirvu A, Riza A, Ioana M, Netea MG (2020). Biomarkers of inflammation and the etiology of sepsis. Biochem Soc Trans.

[CR13] Krasity BC, Troll JV, Weiss JP, McFall-Ngai MJ (2011). LBP/BPI proteins and their relatives: conservation over evolution and roles in mutualism. Biochem Soc Trans.

[CR14] Lambden S, Laterre PF, Levy MM, Francois B (2019). The SOFA score—development, utility and challenges of accurate assessment in clinical trials. Crit Care.

[CR15] Li F, Leier A, Liu Q, Wang Y, Xiang D, Akutsu T (2020). Procleave: predicting protease-specific substrate cleavage sites by combining sequence and structural information. Genomics Proteomics Bioinformatics.

[CR16] Liu YW, Chen CC, Tseng HP, Chang WC (2006). Lipopolysaccharide-induced transcriptional activation of interleukin-10 is mediated by MAPK- and NF-κB-induced CCAAT/enhancer-binding protein δ in mouse macrophages. Cell Signal.

[CR17] Livaditi O, Kotanidou A, Psarra A, Dimopoulou I, Sotiropoulou C, Augustatou K (2006). Neutrophil CD64 expression and serum IL-8: sensitive early markers of severity and outcome in sepsis. Cytokine.

[CR18] Luna-Reyes I, Pérez-Hernández EG, Delgado-Coello B, Ávila-Rodríguez MÁ, Mas-Oliva J (2021). Peptide VSAK maintains tissue glucose uptake and attenuates pro-inflammatory responses caused by LPS in an experimental model of the systemic inflammatory response syndrome: a PET study. Sci Rep.

[CR19] Matsumoto H, Ogura H, Shimizu K, Ikeda M, Hirose T, Matsuura H (2018). The clinical importance of a cytokine network in the acute phase of sepsis. Sci Rep.

[CR20] Miguel-Bayarri V, Casanoves-Laparra EB, Pallás-Beneyto L, Sancho-Chinesta S, Martín-Osorio LF, Tormo-Calandín C (2012). Prognostic value of the biomarkers procalcitonin, interleukin-6 and C-reactive protein in severe sepsis. Med Intensive.

[CR21] Molano Franco D, Arevalo-Rodriguez I, Roqué i Figuls M, Montero Oleas NG, Nuvials X, Zamora J (2019). Plasma interleukin-6 concentration for the diagnosis of sepsis in critically ill adults. Cochrane Database Syst Rev.

[CR22] Nier A, Huber Y, Labenz C, Michel M, Bergheim I, Schattenberg JM (2020). Adipokines and endotoxemia correlate with hepatic steatosis in non-alcoholic fatty liver disease (NAFLD). Nutrients.

[CR23] Ono S, Tsujimoto H, Hiraki S, Aosasa S (2018). Mechanisms of sepsis-induced immunosuppression and immunological modification therapies for sepsis. Ann Gastroenterol Surg.

[CR24] Parlesak A, Schäfer C, Schütz T, Bode JC, Bode C (2000). Increased intestinal permeability to macromolecules and endotoxemia in patients with chronic alcohol abuse in different stages of alcohol-induced liver disease. J Hepatol.

[CR25] Pérez-Hernández EG, Delgado-Coello B, Luna-Reyes I, Mas-Oliva J (2021). New insights into lipopolysaccharide inactivation mechanisms in sepsis. Biomed Pharmacother.

[CR26] Reinhart K, Daniels R, Kissoon N, Machado FR, Schachter RD, Finfer S (2017). Recognizing sepsis as a global health priority—a WHO resolution. N Engl J Med.

[CR27] Rudd KE, Johnson SC, Agesa KM, Shackelford KA, Tsoi D, Kievlan DR (2020). Global, regional, and national sepsis incidence and mortality, 1990–2017: analysis for the Global Burden of Disease Study. Lancet.

[CR28] Schulte W, Bernhagen J, Bucala R (2013). Cytokines in sepsis: potent immunoregulators and potential therapeutic targets—an updated view. Mediators Inflamm.

[CR29] Shao B, Lu M, Katz SC, Varley AW, Hardwick J, Rogers TE (2007). A host lipase detoxifies bacterial lipopolysaccharides in the liver and spleen. J Biol Chem.

[CR30] Singer M, Deutschman CS, Seymour C, Shankar-Hari M, Annane D, Bauer M (2016). The third international consensus definitions for sepsis and septic shock (sepsis-3). JAMA.

[CR31] Thuy S, Ladurner R, Volynets V, Wagner S, Strahl S, Königsrainer A (2008). Nonalcoholic fatty liver disease in humans is associated with increased plasma endotoxin and plasminogen activator inhibitor 1 concentrations and with fructose intake. J Nutr.

[CR32] Van Der Poll T, Van De Veerdonk FL, Scicluna BP, Netea MG (2017). The immunopathology of sepsis and potential therapeutic targets. Nat Rev Immunol.

[CR33] Vreugdenhil ACE, Snoek AMP, Van’T Veer C, Greve JWM, Buurman WA (2001). LPS-binding protein circulates in association with apoB-containing lipoproteins and enhances endotoxin-LDL/VLDL interaction. J Clin Invest.

[CR34] WHO. Service delivery and safety: improving the prevention, diagnosis and clinical management of sepsis. https://www.who.int/news-room/fact-sheets/detail/sepsis (2020). Accessed 23 May 2022.

