# Supplementary material for: The cholesteryl-ester transfer protein isoform (CETPI) and derived peptides: new targets in the study of Gram-negative sepsis
Source: Mol Med. 2022 Dec 19;28:157. doi: 10.1186/s10020-022-00585-3 (PMC9764724; doi:10.1186/s10020-022-00585-3)
Supplement: Supplementary file 1 — Additional file 1: Figure S1. Empirical cumulative distribution of the CETPI measurements. Empirical cumulative distribution of CETPI plasma concentration of controls (n = 47) and patients with infection (n = 50), sepsis (n = 28), and septic shock (n = 27), with a positive blood culture for Gram-negative bacteria. Figure S2. LPS correlates with CETPI plasma levels in patients with Gram-negative bacteraemia. Correlation between LPS with CETPI levels in patients with A infection (n = 31), B sepsis (n = 19), and C septic shock (n = 19). Spearman correlations show associations between LPS and CETPI in patients with infection and sepsis. Table S1. Probability of correlation between cytokines and CETPI in patients with infection shown in Fig. 3I. Table S2. Probability of correlation between cytokines and CETPI in patients with sepsis shown in Fig. 3 J. Table S3. Probability of correlation between cytokines and CETPI in patients with septic shock shown in Fig. 3K. Table S4. Clinical parameters of patients with E, F infection, G sepsis, and H septic shock shown in Fig. 5. [file 10020_2022_585_MOESM1_ESM.pdf]

Additional file 1

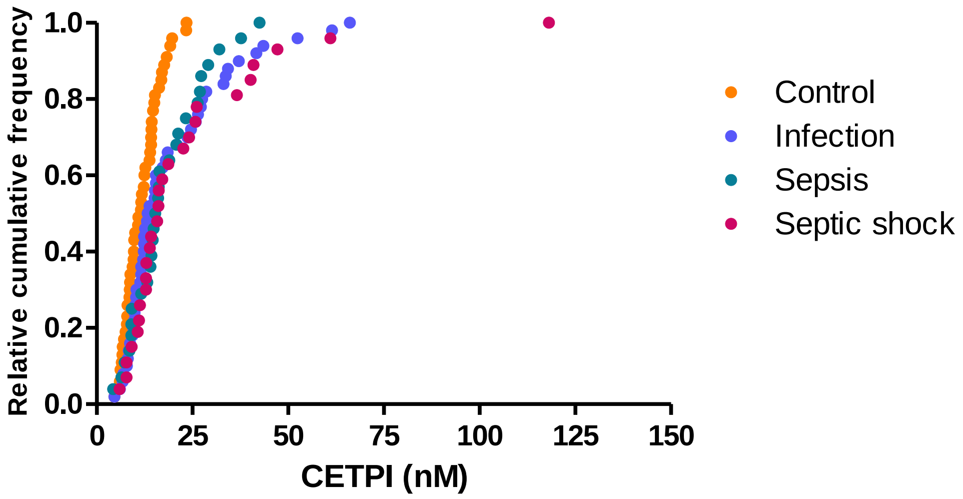

Fig. S1. Empirical cumulative distribution of the CETPI measurements. Empirical cumulative distribution of CETPI plasma concentration of controls (n= 47) and patients with infection (n= 50), sepsis (n= 28), and septic shock (n= 27), with a positive blood culture for Gram-negative bacteria.

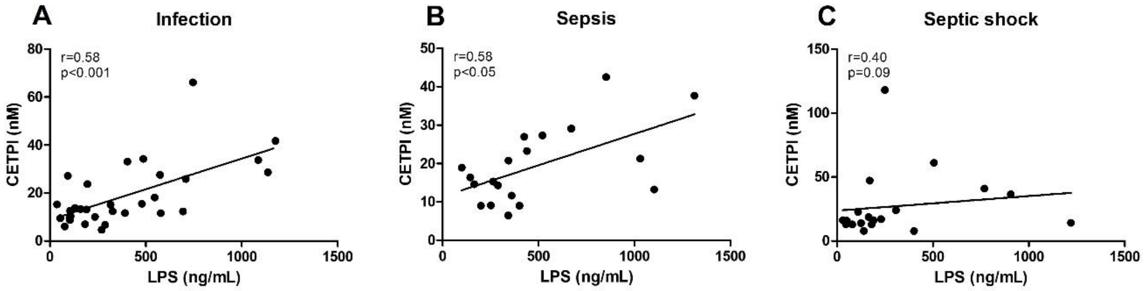

Fig. S2. LPS correlates with CETPI plasma levels in patients with Gram-negative bacteraemia. Correlation between LPS with CETPI levels in patients with **A** infection(n=31), **B** sepsis (n=19), and **C** septic shock (n=19). Spearman correlations show associations between LPS and CETPI in patients with infection and sepsis.

	CETPI	IL1-β	TNF-α	IL-6	IL-8	IL-12p70	IFNy	IL-10
**CETPI**	<.0001	0.1670	0.3311*	0.7530*	0.3869*	0.0941	0.9562	0.1659*
**IL1-β**	0.1670	<.0001	0.8934*	0.0488	0.0423	0.0036	0.1668	0.2375
**TNF-α**	0.3311*	0.8934*	<.0001	0.0048	<.0001	0.1498	0.0382	<.0001
**IL-6**	0.7530*	0.0488	0.0048	<.0001	0.0004	0.3143	0.1029	0.0006
**IL-8**	0.3869*	0.0423	<.0001	0.0004	<.0001	0.1480	0.1418	<.0001
**IL-12p70**	0.0941	0.0036	0.1498	0.3143	0.1480	<.0001	0.0004	0.2184
**IFNy**	0.9562	0.1668	0.0382	0.1029	0.1418	0.0004	<.0001	0.0692
**IL-10**	0.1659*	0.2375	<.0001	0.0006	<.0001	0.2184	0.0692	<.0001

*negative correlation.

Table S1. Probability of correlation between cytokines and CETPI in patients with infection shown in Fig.3 I.

	CETPI	IL1-β	TNF-α	IL-6	IL-8	IL-12p70	IFNy	IL-10
**CETPI**	<.0001	0.5093*	0.9736*	0.8218	0.0960*	0.3767	0.6379*	0.5125*
**IL1-β**	0.5093*	<.0001	0.0251	0.0128	0.0006	0.0218	0.0001	0.0084
**TNF-α**	0.9736*	0.0251	<.0001	<.0001	0.0004	<.0001	0.0007	<.0001
**IL-6**	0.8218	0.0128	<.0001	<.0001	<.0001	0.0048	0.0192	<.0001
**IL-8**	0.0960*	0.0006	0.0004	<.0001	<.0001	0.0473	0.0189	<.0001
**IL-12p70**	0.3767	0.0218	<.0001	0.0048	0.0473	<.0001	0.0010	0.0022
**IFNy**	0.6379*	0.0001	0.0007	0.0192	0.0189	0.0010	<.0001	0.0217
**IL-10**	0.5125*	0.0084	<.0001	<.0001	<.0001	0.0022	0.0217	<.0001

*negative correlation.

Table S2. Probability of correlation between cytokines and CETPI in patients with sepsis shown in Fig.3 J.

	CETPI	IL1-β	TNF-α	IL-6	IL-8	IL-12p70	IFNy	IL-10
**CETPI**	<.0001	0.0042*	0.0978*	0.8463*	0.0460*	0.7298	0.8000*	0.0226*
**IL1-β**	0.0042*	<.0001	0.0019	0.2124	0.0001	0.3738	0.5793	0.0004
**TNF-α**	0.0978*	0.0019	<.0001	0.0035	<.0001	0.4028	0.3965	<.0001
**IL-6**	0.8463*	0.2124	0.0035	<.0001	0.0443	0.9166	0.8019	0.0375
**IL-8**	0.0460*	0.0001	<.0001	0.0443	<.0001	0.3697	0.5608	<.0001
**IL-12p70**	0.7298	0.3738	0.4028	0.9166	0.3697	<.0001	<.0001	0.3160
**IFNy**	0.8000*	0.5793	0.3965	0.8019	0.5608	<.0001	<.0001	0.8029
**IL-10**	0.0226*	0.0004	<.0001	0.0375	<.0001	0.3160	0.8029	<.0001

*negative correlation.

Table S3. Probability of correlation between cytokines and CETPI in patients with septic shock shown in Fig.3 K.

Figure	Age	Sex	Comorbidities	Site of infection	Isolated bacteria	SOFA (previous)	SOFA (T1)
**5E**	65	Female	- Immunosuppression<br>- Dyslipidemia	Urinary tract	*E. coli*	0	0
**5F**	59	Male	- Chronic liver failure<br>- Cancer	Abdominal	*E. coli*	3	4
**5G**	21	Female	- Immunosuppression<br>- Cancer<br>- Dyslipidemia	Abdominal	*E. coli*	4	6
**5H**	55	Male	- Cancer<br>- Endocrinopathy	Abdominal	*E. coli*	2	7

Table S4. Clinical parameters of patients with **E**, **F** infection, **G** sepsis, and **H** septic shock shown in Figure 5.
